# Overcoming cancer risk in inflammatory bowel disease: new insights into preventive strategies and pathogenesis mechanisms including interactions of immune cells, cancer signaling pathways, and gut microbiota

**DOI:** 10.3389/fimmu.2023.1338918

**Published:** 2024-01-15

**Authors:** Haonan Zhang, Yulu Shi, Chanchan Lin, Chengcheng He, Shanping Wang, Qingyuan Li, Yan Sun, Mingsong Li

**Affiliations:** ^1^ Inflammatory Bowel Diseases Research Center, Department of Gastroenterology, Guangdong Provincial Key Laboratory of Major Obstetric Diseases, Guangdong Provincial Clinical Research Center for Obstetrics and Gynecology, The Third Affiliated Hospital of Guangzhou Medical University, Guangzhou, China; ^2^ Department of Oncology, Nanfang Hospital, Southern Medical University, Guangzhou, Guangdong, China; ^3^ Department of Gastroenterology, Quanzhou First Hospital Affiliated to Fujian Medical University, Quanzhou, Fujian, China; ^4^ Guangdong Provincial Key Laboratory of Gastroenterology, Department of Gastroenterology, Nanfang Hospital, Southern Medical University, Guangzhou, Guangdong, China

**Keywords:** inflammatory bowel disease, inflammation associated cancer, cancer risk, immune cells, cancer prevention

## Abstract

Inflammatory bowel disease (IBD), characterized primarily by gastrointestinal inflammation, predominantly manifests as Crohn’s disease (CD) and ulcerative colitis (UC). It is acknowledged that Inflammation plays a significant role in cancer development and patients with IBD have an increased risk of various cancers. The progression from inflammation to carcinogenesis in IBD is a result of the interplay between immune cells, gut microbiota, and carcinogenic signaling pathways in epithelial cells. Long-term chronic inflammation can lead to the accumulation of mutations in epithelial cells and the abnormal activation of carcinogenic signaling pathways. Furthermore, Immune cells play a pivotal role in both the acute and chronic phases of IBD, contributing to the transformation from inflammation to tumorigenesis. And patients with IBD frequently exhibit dysbiosis of the intestinal microbiome. Disruption of the gut microbiota and subsequent immune dysregulation are central to the pathogenesis of both IBD and colitis associated colorectal cancer (CAC). The proactive management of inflammation combined with regular endoscopic and tumor screenings represents the most direct and effective strategy to prevent the IBD-associated cancer.

## 1 Introduction

Inflammatory bowel disease (IBD) is a chronic inflammatory disorder of unclear etiology and unknown mechanisms, primarily involving inflammation of the gastrointestinal tract. The two most common forms are Crohn’s disease (CD) and ulcerative colitis (UC) (1). Patients with IBD have an increased risk of developing gastrointestinal tumors. In addition, some extraintestinal malignancies are also associated with IBD (2). Key risk factors for tumor development in IBD patients include early onset of IBD (younger than 15 years), colonic-type CD, familial history of colorectal cancer (CRC), and IBD-associated complications, such as foreshortened colon, strictures, inflammatory pseudopolyps, and primary sclerosing cholangitis (3). And CRC caused by IBD often presents with more low-differentiated tumors, which are more aggressive, leading to a poorer prognosis (4). Persistent hyperactive and uncontrolled inflammation can lead to severe complications for IBD patients, notably carcinogenesis. Unlike sporadic CRC, which originates from adenoma transformation, IBD-associated cancers undergo a progression from inflammation to dysplasia and then to tumor formation. In IBD, clonal evolution begins long before evident tumor formation and may be accelerated by the repetitive cycle of epithelial damage and repair, which is characteristic of the colitis associated colorectal cancer (CAC) (5). The pathogenesis of IBD-associated cancer is believed to be a result of a combination of environmental, genetic, microbial, and immunological factors (6). This review will delve into the incidence, mechanisms, and preventive and therapeutic measures of various tumors in the context of IBD. In addition, this review rationalizes the seemingly contradictory dual role of some kinds of immune cells in inflammatory cancers.

## 2 The risk of cancer associated with IBD

### 2.1 Colorectal and anal cancer

Chronic UC is considered a risk factor for CRC. The risk of developing CRC from long-standing Crohn’s disease related colitis is believed to be similar to that of UC (7). A retrospective study conducted in China between 2000 and 2012 documented 642 cases, revealing the identification of four cases of CRC associated with UC. The overall cancer risk in this study was found to be 0.64%. In UC-related CRC patients, the median duration of UC was 15.5 years with 75% of them being diagnosed at an advanced stage (8). A meta-analysis involving 31,287 ulcerative colitis patients reported 293 cases of CRC. Using a pooled prevalence analysis from various studies, the overall prevalence was 0.85%. The risk of CRC at 10 years was 0.02%, increasing to 4.81% at 20 years, and further to 13.91% at 30 years (9). Another meta-analysis, which included 25 studies comprising 8,034 IBD-CRC patients and 810,526 non-IBD CRC patients, found that the overall survival (OS) for IBD-CRC patients was significantly poorer than that for non-IBD patients, with a hazard ratio (HR) of 1.33. While the OS for ulcerative colitis-associated CRC was better than that for Crohn’s disease-associated CRC (HR=0.79). To summarize, IBD-associated CRC patients had a lower rate of R0 resection with a odds ratio (OR) of 0.6 compared to non-IBD-CRC patients (10).

A retrospective study from New Zealand found that patients with colon-type CD have a significantly increased risk of developing CRC. The study collected clinical data from 649 CD patients, of which 436 had ileocolonic or colon-type CD. Among them, 13 were diagnosed with CRC, resulting in an overall cancer risk of 2.98%. The median age at diagnosis of CRC was 58.5 years, and the average duration of CD before the cancer diagnosis was 20.4 years. Patients with colon CD have a significantly elevated risk of CRC compared to the general population (11).

Patients diagnosed with IBD, particularly CD frequently, often experience perianal complications alongside intestinal inflammation. A recent meta-analysis revealed a higher incidence of anal cancer in patients with perianal CD compared to the general patient population. Notably, perianal involvement accounted for the majority of cases of anal cancer, representing 46% of the cases (12). Another meta-analysis highlighted an increased risk of anal cancer in patients diagnosed with both CD and UC. The summarized incidence rates (IRs) were 6 (3-11) for CD and 3 (2-4) for UC (13).

In summary, CRC arising from IBD exhibits a higher incidence and poor prognosis when compared to sporadic CRC, while CRC originating from colon-type CD appears to have a higher incidence and worse prognosis than that originating from UC.

### 2.2 Small bowel cancer

In patients with IBD, particularly CD, there is an increased incidence of small bowel cancer (SBC), with small bowel adenocarcinoma being the most common type of SBC (14).

It is typically found in the narrowed or inflamed ileal regions. In a population-based cohort study from both Sweden and Denmark, among 168,896 IBD patients (CD: 47,370; UC: 97,515; Unclassified IBD: 17,011), 237 IBD patients were diagnosed with SBC during the follow-up period (CD: 24.4 per 100,000 person-years; UC: 5.88 per 100,000 person-years). In contrast, out of a control group of 20,399,257 people, 640 were diagnosed (equating to 2.81 per 100,000 person-years and 3.32 per 100,000 person-years, respectively). The relative risk of SBC-related mortality is increased in both CD and UC patients (15).

A comprehensive meta-analysis encompassing 26 studies revealed a significant association between IBD and a 67% increased risk of combined gastric, small bowel, and CRCs. Notably, the predominant increase in risk was observed in SBC, with gastric cancer being the exception. Furthermore, CD notably increased the risk for both small and large bowel cancers, while UC primarily raised the risk for CRC alone (16).

### 2.3 Gastric cancer

In the general population, H. pylori infection is a significant risk factor for gastric cancer. However, several studies have reported a notably lower prevalence of this infection in IBD patients. This phenomenon may be attributed to the prolonged use of anti-inflammatory medications in IBD patients (17, 18).

A Danish study revealed that IBD patients, particularly those with CD, had an increased risk of CRC, SBC, and both gastric (incidence ratios (SIRs) =1.2) and extraintestinal (SIRs=1.3) malignancies (19). And a study from Japan revealed a significantly increased risk of CRC (SIRs=5.8) and gastric cancer at (SIRs=1.86) when compared to the general population (20). However, another study from Japan indicated no discernible difference in gastric cancer risk between CD patients and the general population, while the risk for CRC and leukemia was considerably elevated compared to the general populace (21). But patients with gastric cancer and CD may experience significantly reduced survival rates compared to the general population (22). The link between gastric cancer and IBD remains unclear. Further research into its incidence and pathogenesis is essential.

### 2.4 Extragastrointestinal cancer

Inflammation is a critical mediator in the process of carcinogenesis. In addition to its effects on the gastrointestinal tract, inflammatory bowel disease often presents in extragastrointestinal organs. Recent research has identified associations between specific immune-mediated disorders and an increased risk of cancers in distant organs. One particular study documented an increased risk of extraintestinal cancers in patients with CD with an Incidence Rate Ratio (IRR) of 1.43 and in those with UC with an IRR of 1.15 such as skin malignancies, hepatobiliary cancers, hematologic malignancies and lung cancer.

For instance, one study found that individuals with Crohn’s disease exhibit a significantly increased risk of liver cancer with a HR of 4.01, while those with ulcerative colitis demonstrate an enhanced risk with an HR of 2.59 (23–25).

Primary sclerosing cholangitis is the classic hepatobiliary manifestation of inflammatory bowel disease, often exhibiting a chronic and progressive course. It is characterized by a gradual fibroinflammatory deterioration of the intrahepatic and/or extrahepatic bile ducts. Notably, patients diagnosed with this condition exhibit a considerably heightened risk of malignancy compared to the general population (26). An epidemiological study found an association between UC and intrahepatic cholangiocarcinoma, with an OR of 1.87 (27). A Mendelian randomization study showed that in East Asia, individuals diagnosed with IBD exhibited a 1.28-fold increase (p = 0.0065) in the incidence of hepatocellular liver cancer (HLC) compared to the general populace. Furthermore, patients suffering from UC presented with a 1.12-fold (p < 0.0001) elevated incidence of hepatocellular carcinoma (HCC) and a 1.31-fold (p = 0.0027) heightened incidence of cholangiocarcinoma (CCA) (28).

Besides, Some evidence from extensive multicenter studies and meta-analyses robustly indicates an increased risk of prostate cancer in patients with IBD, particularly in those with UC. And CD is also notably associated with an increased risk of renal cancer. But there doesn’t seem to be a significant correlation between IBD and the incidence of breast cancer (29–32).

Due to the specific nature of IBD, patients commonly exhibit chronic intestinal inflammation along with multiple organ involvement and increasing their risk of developing various cancers. Thus, it is of great significance to explore the pathogenesis of IBD-associated cancers. Presently, research mainly focuses on CAC, while research regarding the pathogenesis of other IBD-associated cancers remains scarce. Future studies exploring the pathogenesis of the other IBD-associated cancers will not only enhance our understanding of the mechanisms underlying IBD-related cancers but will also help to unravel how IBD involves extraintestinal organs and the mystery of its pathogenic origin.

## 3 The mechanisms of cancer associated with IBD

### 3.1 Immune cells

Inflammation is the immune system’s response to injury, with immune cells participating actively in both acute and chronic inflammatory phases of IBD. These cells are instrumental in the progression from chronic inflammation to tumorigenesis. The immune system comprises a diverse array of cell types, each with specialized functions that work collaboratively to defend against external threats. This section will elucidate the types of immune cells involved in the development of inflammation and their potential role in oncogenic transformation within the context of IBD (33).

### 3.2 Macrophages

Macrophages, integral components of the innate immune system, primarily arise from monocytes. When stimulated by cytokines and microbial agents, they undergo functional specialization and polarization. These polarized macrophages can be broadly categorized into two distinct types: M1 and M2, each assigned specific functions (34). M1 macrophages exhibit pronounced proinflammatory and antimicrobial activities, whereas M2 macrophages exhibit robust phagocytic capabilities, which facilitate the clearance of debris and apoptotic cells and possess anti-inflammatory properties (35). But during tumorigenesis, macrophages also play dual roles, both anti-cancer and pro-tumor. Specifically, M1 macrophages enhance tumor immunity, while M2 macrophages, a principal constituent of tumor-associated macrophages (TAMs), promote tumorigenesis and metastasis (36). Considering that inflammatory carcinogenesis is driven by chronic inflammation, and macrophages play dual roles in both inflammation and cancer, questions have arisen regarding the potentially contradictory functions of these macrophages in the development of IBD-associated cancers.

Chronic inflammatory stimulation is a primary contributor to IBD-associated cancers. Some studies suggest that during the early stages of CAC development, there is an upregulation of Xanthine oxidoreductase (XOR). This upregulation may drive the polarization of M1 macrophage, thereby shaping the tumor microenvironment to favor CAC progression (37). Low doses of Diphenyleneiodonium (DPI) mitigate intestinal inflammation by decreasing macrophage recruitment and suppressing M1 macrophage activation (38). In the early CAC development, Dihydroartemisinin (DHA) curtails macrophage activation and infiltration in the colonic mucosa via the TLR4 signaling pathway, consequently reducing pro-inflammatory cytokine expression. In contrast, during the advanced stages of CAC, DHA impedes tumor growth by inducing tumor cell cycle arrest and apoptosis. And thalidomide treatment impedes M1 polarization within the inflammatory microenvironment, reduces DSS-induced colonic inflammation, facilitates mucosal healing, and curtails the progression of CAC (39).

M2 macrophages play a crucial role in mitigating intestinal inflammation. Some researchers have developed colon-accumulated gold nanoclusters that target and augment M2 macrophages, consequently attenuating the progression from IBD to CAC through an Nrf2-dependent pathway (40). In the CAC microenvironment, exosomal miR-93-5p secreted by G-MDSC facilitates the differentiation of M-MDSC into M2 macrophages, thereby promoting the development of CAC (41). IL-6 promotes the polarization of macrophages towards the tumor-promoting M2 phenotype, which, in turn, produces the chemokine CCL-20. Subsequently, CCL-20 enhances CAC progression by selectively recruiting CCR-6-expressing B-cells and γδ T-cells (42).

In summary, M1 and M2 macrophages play distinct yet antagonistic roles during different stages of CAC. In the early stages of CAC development, which coincide with the early phase of IBD, persistent overactivation of M1 macrophage and continuous pro-inflammatory responses lead to tissue damage and increased risk of carcinogenesis. In the later stages of CAC development, although M2 macrophage can alleviate inflammation by promoting tissue repair, they simultaneously foster a tumor microenvironment conducive to immune cell functional tolerance, thereby creating favorable conditions for tumor growth (43). Therefore, there is a dynamic imbalance between M1 and M2 macrophages during the progression of CAC. In the early stages, M1 macrophages may suppress the function and survival of M2 macrophages through oxidative stress mechanisms. Additionally, there is competition between them for cytokines and chemokines (44). However, in the later stages of CAC progression, the establishment of a tumor immune tolerance microenvironment, recruitment and activation of Treg cells, and inhibition of M1 macrophages’ anti-tumor immune responses occur. M2 macrophages can also inhibit the function of M1 macrophages by affecting the STAT3 and PI3K/AKT pathways (45).

### 3.3 T cells

#### 3.3.1 CD4 and CD8 T cells

T cells, a subset of lymphocytes, can be classified based on their T-cell receptors (TCRs) into either αβ or γδ subsets. Notably, the αβ T cells constitute the predominant subset of the T cell repertoire and encompass distinct populations, including CD4 and CD8 T cells (46). In patients with IBD, activated CD4+ and CD8+ T cells, present in both the peripheral blood and intestinal mucosa, play a pivotal role in mediating the inflammatory response (47, 48). It has been shown that the knockdown of CerS4 in T cells has been demonstrated to lead to prolonged activation of both T cell responses and the NF-κB signaling pathway.This, in turn, contributes to the progression of CAC (49).

CD8 T cells are often considered indispensable in the fight against tumor growth and are conventionally regarded as the primary immune effectors for targeting and combating cancer cells (50), relying on signals from CD4+ T cells (51). Infiltration and function of CD8 T cells in the tumor microenvironment determine resistance to tumorigenesis (52). IL-37 has been shown to increase CAC through CD8 T cell inactivation (53). Dysfunction in the Atg7 autophagy gene within intestinal epithelial cells (IECs) results in the significant accumulation of T cells, particularly CD8+ T lymphocytes, in the colonic lamina propria, thereby impeding the progression of CAC (54). While CD8 T cells typically inhibit tumor formation, one cannot help but wonder whether there are also potential drawbacks to the overactivation of CD8 in the specific context of tumors caused by inflammation in IBD. Interestingly, studies have indicated that an appendectomy may alleviate colorectal inflammation in patients with UC by reducing CD8 T cells infiltration. However, this is concomitantly associated with a heightened risk of CAC (55).

CD4+ T cells which are closely associated with the development of IBD-associated inflammation can be further delineated into regulatory and effector T cells (56). The dysregulated expression of the tumor suppressor gene p27, may indirectly facilitate the progression of gastrointestinal epithelial malignancies. This is postulated to occur through the increased production of inflammatory mediators from a spontaneously proliferating subset of CD4+ effector memory T cells (57).

Effector T cells, on the other hand, can be categorized into Th1, Th2, and Th17 subsets, each secreting pivotal cytokines. Th1 cells produce cytokines such as TNF-α, IFN-γ, and IL-6, which facilitate the recruitment of macrophages to inflammation sites and are implicated in the formation of CD granulomas. Th17 cells produce IL-17, IL-22, and IL-21, and in conjunction with T1 cells, they contribute to the inflammatory cascade in CD, initiating phenomena like transmural inflammation (58, 59). Conversely, Th2 cells primarily contribute to UC-associated inflammatory processes by secreting IL-4, which has implications in UC’s intestinal mucosal inflammation (60).

In the context of CAC, some studies argue that Th1 and Th2 cells exhibit contrasting roles. While Th1 cells seem to provide protective effects, Th2 cells are associated with tumor promotion (61, 62). Some other studies have found that patients with active IBD exhibit elevated levels of TNF-α and IFN-γ in the inflamed colon. TNF-α enhances ETS-1 expression and augments Th1-mediated mucosal inflammation, contributing to the progression of CAC through the mediation of CIRBP (63).

Regulatory T (Treg) cells are pivotal in sustaining immune homeostasis and thwarting autoimmunity. In oncology, an accumulation of Tregs is typically correlated with an unfavorable prognosis. However, various subpopulations of Tregs exist, each potentially exerting distinct effects on tumor progression (64). Foxp3+ Treg cells modulate and inhibit a wide range of both innate and adaptive immune responses (65). In the CAC model, the transient depletion of Foxp3+ Treg cells during tumor progression results in suppressed tumor growth and dissemination. This phenomenon is associated with an augmented presence of CD8 T cells producing IFNγ and granzyme B (66). STAT6 can facilitate the progression of CAC by suppressing the function of Foxp3+ Treg cells (67). However, during inflammation and early dysplasia, there is a notable expansion of RORγt+ Treg cells. This expansion in IBD is associated with the activation of Wnt-β-catenin signaling, leading to the co-expression of numerous pro-inflammatory cytokines that foster tumorigenesis (68).

#### 3.3.2 NKT cells

NKT cells have been implicated in the pathogenesis of IBD. Their maturation relies on the thymus, with a significant proportion deriving from CD4+CD8+ double-positive (DP) thymocytes (69). These cells display surface markers characteristic of both T cells (such as TCR and CD3) and NK cells (including NKG2D and CD161). Based on TCR variances, they can be classified into NKT type 1 and NKT type 2 cells.

Observations indicate a diminished presence of NKT type 1 cells in both the intestinal tissue and peripheral blood of IBD patients. Conversely, a notable accumulation of NKT type 2 cells has been reported in the intestinal tissues of UC patients (70). NKT type 2 cells may exacerbate UC through secreting IL-13, a cytokine known to induce apoptosis in intestinal epithelial cells and compromise the intestinal mucosal barrier. In the lamina propria of CD patients, NKT type 1 cells can produce pro-inflammatory cytokines like TNF-α, IFN-γ, and IL-13, further contributing to mucosal barrier disruption (71, 72). Interestingly, some studies suggest that NKT type 1 cells may provide protection against colitis in mouse models by secreting IL-9 (73). In summary, NKT type 1 cells exhibit both protective and pathogenic tendencies in IBD, while NKT type 2 cells lean more towards promoting intestinal inflammation (58).

#### 3.3.3 γδT cells

γδT cells can be primarily classified into two subpopulations: Vδ1 T cells and Vδ2 T cells. In healthy tissues, Vδ1 T cells constitute the dominant γδT cell subset. However, in the context of chronic IBD, there is a significant enrichment of Vδ2 T cells. These cells produce higher levels of cytokines such as IFN-γ, TNF-α, and IL-17 in chronic inflammatory conditions compared to Vδ1 T cells. This observation underscores the potential role of Vδ2 T cells in the pathogenesis of both IBD and CAC (74).

### 3.4 Neutrophils

During an inflammatory response, neutrophils not only accumulate but also become activated, leading to the release of reactive oxygen species (ROS), various cytokines, and other inflammatory mediators. These components then interact through specific signaling pathways, orchestrating a cascade of responses that regulate both anti-inflammatory and pro-inflammatory mechanisms, maintaining homeostatic balance within the human body (75).

A growing body of evidence underscores the dual role of neutrophils. In addition to their well-known pro-inflammatory functions, certain neutrophil subpopulations demonstrate anti-inflammatory properties. These neutrophils can self-limit their chemotaxis through selective cytokine secretion, facilitate the clearance of pro-inflammatory cells, and significantly contribute to tissue repair and regeneration processes (76). Specifically, CD177+ neutrophils enhance bactericidal activity and produce IL-22, thereby exerting a protective influence in IBD (77). Neutrophils undergoing apoptosis can modulate their chemotaxis through the activation of macrophages and the subsequent release of pertinent cytokines. This process facilitates their own clearance, culminating in the attenuation and resolution of inflammation (78).

Besides their contribution to the inflammatory processes in IBD, research indicates that neutrophils release free radicals and carcinogenic entities, including N-nitroso compounds. This secretion heightens the susceptibility to cancer among IBD patients (79, 80).

### 3.5 Innate lymphoid cells

Innate lymphoid cells (ILCs) play a pivotal role in modulating intestinal inflammation and the pathogenesis of IBD. Derived from common lymphoid progenitors (CLPs), ILCs are classified into three primary groups: Group 1, which includes NK cells and ILC1; Group 2, represented by ILC2; and Group 3, encompassing ILC3 (81).

#### 3.5.1 NK cells

In IBD patients, there is an observed elevation in the number of NK cells within the lamina propria. These cells may contribute to the pathogenesis of IBD by secreting interferon gamma, thereby promoting the differentiation of T1 cells from naïve CD4+ T cells. Furthermore, the excessive presence of interferon gamma has a detrimental impact on tight junctions in the intestinal mucosal barrier (82), which is a crucial event that exacerbates chronic inflammation in IBD and subsequently triggers the development of CAC. Contrarily, several studies employing animal models of colitis have demonstrated a protective role of NK cells against the development of colitis. This protection is mediated via the inhibition of NKG2A receptor and direct cell-cell interactions, resulting in the attenuation of pro-inflammatory activities exhibited by neutrophils, including the secretion of cytokine and ROS (83).

#### 3.5.2 ILC1 cells

ILC1 cells possess the capability to secrete interferon gamma and express the transcription factor, T-bet. Under the influence of IL-12, ILC1 cells can differentiate from ILC3 cells, specifically the RORγt(+) ILC3 subtype. Notably, there is a reported upsurge in the prevalence of ILC1 cells within the inflamed intestine of Crohn’s disease patients (84). Similarly, a heightened frequency of ILC1 has been observed in the dysplastic intestinal tissue of ulcerative colitis patients. An analysis leveraging publicly available single-cell RNA sequencing (scRNA-seq) data for CD and CRC revealed a significant enrichment of CD-inducible genes. This enrichment was notably observed in ILC1, which are known to promote the development of CRC through their pro-inflammatory functions. Furthermore, a significant enrichment of these genes was identified in IBD-associated tumors (85). Collectively, these findings underscore a potential role for ILC1 in sustained intestinal inflammation and carcinogenesis (86).

#### 3.5.3 ILC2 cells

In IBD patients, an increased presence of ILC2 cells has been observed within diseased tissues (86). These cells are proficient in secreting IL-13 and IL-5, acting as principal contributors of T2 cytokines (82). ILC2 cells appear to play a crucial role in maintaining the structural integrity of the intestinal mucosal barrier. Notably, IL-13, produced by these cells, appears to facilitate the differentiation of intestinal stem cells into goblet and Tuft cells, which are essential for rectifying intestinal damage (87). IL-33, which can be released from compromised epithelial cells, appears to be significant for ILC2 cells in the pathogenesis of IBD (88). However, there is a discrepancy in findings across various studies. One study indicated that a deficiency in IL-33 hindered the differentiation of ILC2 and Th17 cells, thereby attenuating cytokine levels, such as IL-6 and IL-1. This, in turn, protected mice from DSS-induced colitis, with the study also pointing out that external introduction of IL-33 worsened colitis (89). Conversely, another research found that IL-33 offered protection against DSS-induced colitis by bolstering the proliferation of ILC2 and Treg cells (90). Consequently, the precise mechanistic role of ILC2 cells in the pathogenesis of IBD requires further elucidation. Given the suggested involvement of IL-33 in the progression from colorectal adenomas to CRC, it becomes imperative to investigate the role of ILC2 cells in the development of CAC (91).

#### 3.5.4 ILC3 cells

ILC3 cells predominantly segregate into two subtypes: NKp44+ILC3s and NKp44-ILC3. Within the intestinal lamina propria, the majority of the ILC cell population is comprised of NKp44+ILC3s cells. Notably, research has indicated a reduced frequency of these cells in the affected intestinal tissues of IBD patients. Functionally, these cells can produce IL-22, a cytokine that fortifies the integrity of the intestinal mucosal barrier and stimulates the production of antimicrobial agents (92). However, the enhancement of the STAT3 signaling cascade by IL-22 in epithelial cells augments proliferation, implicating its potential role in CRC progression. In patients with CD, NKp44+ICL3 cells in affected intestinal tissues seem to produce decreased amounts of IL-22 while retaining the capability to produce IFN-γ. By secreting GM-CSF, these cells also amplify the recruitment of pro-inflammatory monocytes (82, 86). While their involvement in the pathogenesis of IBD and CAC is evident, the exact roles of these cells require more comprehensive investigations.

**Figure 1:**
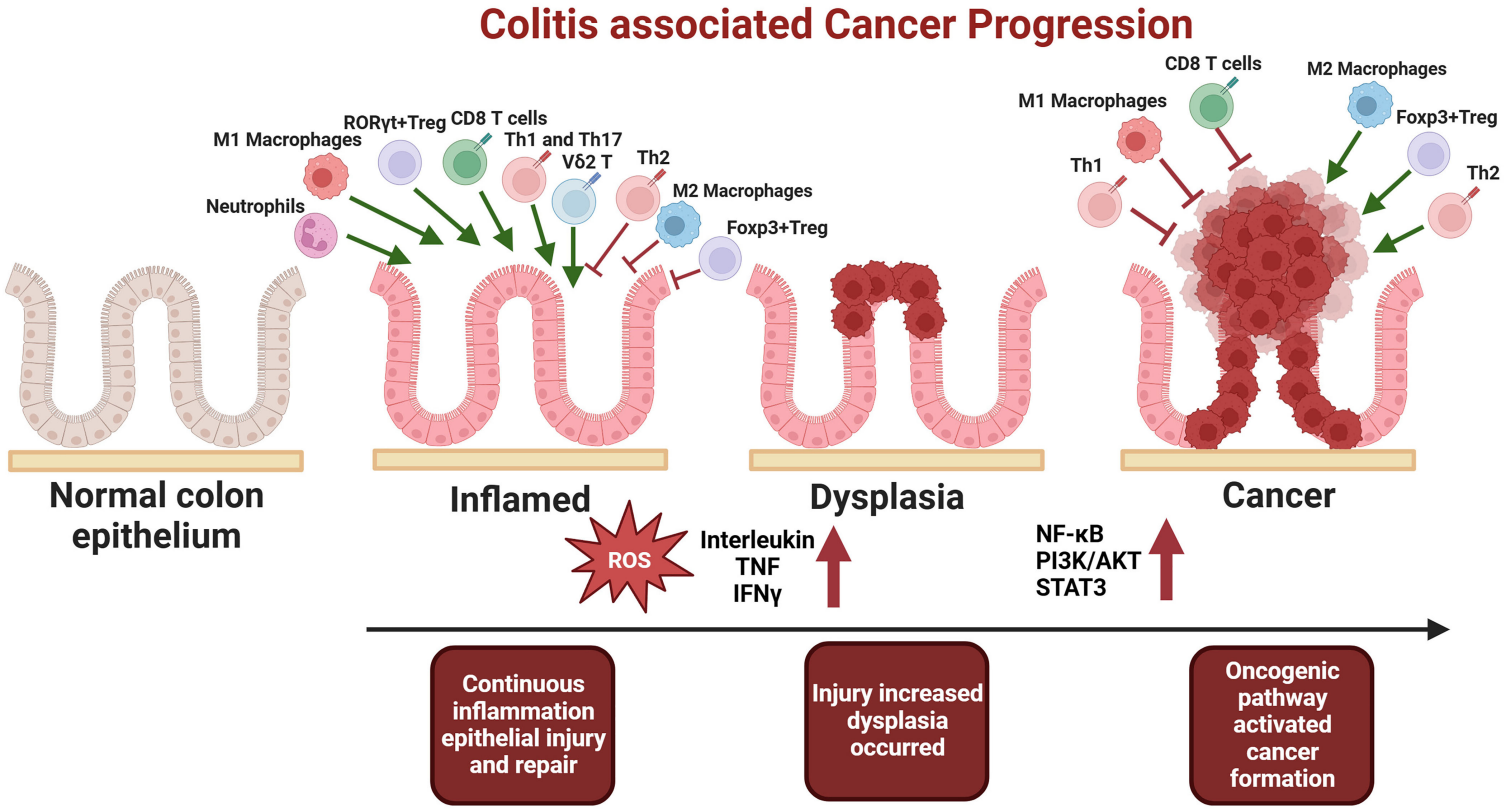
In the early stages of tumorigenesis, there is an increase in pro-inflammatory cells, including M1 macrophages, CD8 T cells, Th1, Th17, neutrophils, Vδ2 T cells, and RORγt+ Treg cells. Concurrently, there is a decrease in suppressive cells, such as M2 macrophages, Th2 and Foxp3+ Treg. This imbalance results in excessive pro-inflammatory cytokine secretion, heightened inflammation, damage to epithelial cells, and the subsequent mutation of normal epithelial cells, ultimately leading to dysplasia and inflammation-associated carcinogenesis. Conversely, after the establishment of the tumor microenvironment, immunosuppressive cells, namely M2 macrophages, Foxp3+ Treg, and Th2, become dominant, facilitating tumor immune evasion and progression to advanced stages

The prevailing view suggests that the tumor microenvironment should lean towards immune suppression. So how to activate the immune responses within the tumor is crucial for the efficacy of immunotherapies. However, since inflammation is also a form of immune response, the hyperactivation of the immune system often aids the progression of inflammation-associated cancers. Current researches indicated that in the early stages of inflammation-to-cancer transformation, the damage to epithelial cells caused by inflammation is the primary driving force. But after tumour formation, it tends to shift the microenvironment towards immune suppression to evade immune cell attack. This also clarifies some apparent contradictions in the role of immune cells with dual functions in the mechanisms of inflammation-associated tumor development (**Figure 1**). Therefore, future explorations using multi-omics technologies to investigate the crosstalk between these immune cells and to identify key cellular subgroups in IBD-related tumors as potential therapeutic targets are of paramount importance.

## 4 Signaling pathway

Unlike the occurrence of sporadic cancers, the development of tumors in IBD follows the inflammation-dysplasia-cancer sequence. The persistent stimulation of epithelial cell proliferation in an inflammatory environment is considered crucial in the etiology of tumors in individuals with IBD, This underscores the pivotal relationship between chronic inflammation and tumorigenesis in these conditions (93).

Cancers developed against the backdrop of IBD exhibit distinct molecular characteristics depending on their locations. For instance, patients with Crohn’s disease face an increased risk of adenocarcinoma and neuroendocrine tumors in small bowel. Most patients with IBD-SBC have active moderate to severe IBD. Unlike IBD-associated colorectal adenocarcinoma, IBD-SBC does not exhibit evidence of microsatellite instability in tumors, highlighting the heterogeneity in molecular features of cancers associated with IBD (94). A connection between IBD and Hepatocellular Carcinoma (HCC) is also recognized; although the precise mechanisms remain to be elucidated. Current understanding suggests associations with molecules such as CXCL2, MMP9, SPP1, and SRC, underscoring the need for further investigative studies to clarify these relationships (95).

Classical signaling pathways, including NF-κB, PI3K/AKT, and STAT3, are crucially involved in the manifestation of inflammation and the onset of CAC. Their significance in understanding IBD-related oncological developments. The roles of these pathways in CAC will be discussed in detail below.

### 4.1 NF-κB

Nuclear Factor-kappa B (NF-κB) is a pivotal transcription factor involved in numerous physiological processes, including inflammation, stress response, cellular differentiation, proliferation, and apoptosis, and is significantly correlated with tumor initiation and progression. This underlines its substantial impact on cellular and molecular biology and its critical role in understanding and addressing various pathological conditions.

NF-κB plays an instrumental role in augmenting the production of pro-inflammatory cytokines, adhesion molecules, and chemotactic factors and in modulating the activity and development of immune cells. It induces the maturation of dendritic cells and the formation of memory T cells, influences macrophages to release abundant pro-inflammatory agents and polarize to M1 phenotype, and directs neutrophils to anti-apoptotic states and inflammatory sites. These roles highlight the significant implications of NF-κB in immunological responses, inflammatory processes, and potential therapeutic interventions for inflammatory diseases (96, 97).

Pro-inflammatory agents, such as TNF-α, IL-1, and IL-6, which are encoded by the NF-κB signaling pathway, play a crucial role in both the pathogenesis of inflammation-induced tissue damage and the promotion of tumorigenesis. Specifically, TNF-α can induce cellular transformation by stimulating the generation of reactive oxygen species and facilitating DNA damage, These findings illuminate the pathway’s significant implications in tumorogenesis and inflammatory conditions (98). In patients with IBD and CRC, there is an upregulation of PIK3R3 in intestinal epithelial cells. This heightened PIK3R3 expression triggers the activation of the NF-κB pathway, resulting in a subsequent decrease in ZO-1 expression, Consequently, this molecular cascade increases the susceptibility of IBD patients to cancer development (99). Mice lacking RNF138 exhibit a marked increase in NF-κB signaling and demonstrate a heightened susceptibility to the transition from colitis to invasive malignant tumors (100). The interplay between the gut microbiota and intestinal epithelial cells play a crucial role in carcinogenesis associated with IBD. Fusobacterium nucleatum activates the Toll-like receptor 4 signaling pathway leading to MYD88, resulting in the activation of NF-κB and increased expression of miR21. This elevates the risk of CAC onset in patients and is linked to a less favorabler prognosis (101). Besides its the expression of the NF-κB pathway in epithelial cells, its role in macrophages is also significant in CAC. MiR-148a directly targets several established upstream regulators of NF-κB and STAT3 signaling pathways, including GP130, IKKα, IKKβ, IL1R1, and TNFR2. This modulates the activation of NF-κB and STAT3 in macrophages and colonic tissues, thereby influencing the onset of colitis and colitis-associated tumorigenesis (102).

### 4.2 PI3K/AKT

PI3K, a phosphoinositide kinase, is involved in various cellular signaling pathways. Serine/threonine-protein kinase B (AKT) is a member of the AGC kinase family and is regulated by growth factors. The product of PI3K activation interacts with the pleckstrin homology domain of AKT, leading to its translocation to the plasma membrane and subsequent activation through phosphorylation by upstream kinases such as PDK1. AKT plays a role in numerous cellular functions, including survival, proliferation, growth, glucose metabolism, apoptosis, angiogenesis, transcription, and migration (103).

The activation of the PI3K/AKT signaling cascade augments the synthesis and secretion of the pro-inflammatory cytokine TNF-α, inducing a cytokine disequilibrium and manifesting as inflammatory responses via a series of molecular interactions (104). Studies indicate that the PI3K/AKT signaling pathway synergizes with the Wnt pathway to amplify β-catenin signaling during inflammatory responses. In the progression from UC to CAC, the PI3K-induced and AKT-mediated β-catenin signaling is pivotal for the activation of progenitor cells. These elements can be identified as biomarkers for aberrant colon developmental transitions. Notably, not only in the colon epithelial cells, but also the activation of the Wnt/β-Catenin pathway in T-effector cells, particularly T17 and Treg cells, contributes to CAC development as well (105). Reducing PI3K/AKT signaling pathways can lead to a decrease in colonic immune cell infiltration, significantly inhibiting the occurrence of colitis and intestinal tumors (106).

### 4.3 STAT3

STAT3 serves as a pivotal transcription factor involved in inflammation and cellular growth, with a crucial role in modulating cell apoptosis. In patients with CAC, there is a significant increase in the activation levels of STAT3. With the activation of STAT3, anti-apoptotic genes including BCL2 and BCL-XL are activated. This means tumor invasion, metastasis, and poor prognosis in CAC (107).

Key inflammatory mediators, encoded by NF-κB target genes and prominently exemplified by IL-6 and IL-22, which have a central position in orchestrating diverse immune responses throughout IBD pathogenesis (108). And Located in the cytoplasm, STAT3 responds to inflammatory cytokines, particularly IL-6 and IL-22 (109). A wealth of studies underscores the indispensability of IL-6 and STAT3 for intestinal epithelial cell viability and CAC progression. Upon IL-22 and IL-6 stimulation in epithelial cells, the activation of the STAT3 pathway not only enhances cell viability but also suppresses suppressing apoptosis, underscoring its significance in the transition from IBD to cancer (110, 111). Their inhibition curtails tumor emergence in CAC (112, 113). The Notch pathway is considered a downstream effector of the IL-6/STAT3 axis. It is pivotal in regulating the self-renewal and differentiation of normal cells across various tissues, also guides the self-renewal and tumorigenic potentials of human cancer stem cells (114).

The signaling pathways leading to CAC predominantly target the NF-κB, PI3K/AKT, and STAT3 pathways, which are critically associated with both inflammation and cancer. Therefore, therapeutic agents targeting these pathways merit clinical validation for their efficacy in CAC treatment. Given the challenges in acquiring clinical samples of CAC, current mechanistic studies are largely confined to cellular and animal models. In the future, collecting samples from CAC patients at various stages, from inflammation to dysplasia and then to cancer, and conducting single-cell sequencing will be crucial to elucidate the molecular mechanisms that distinguish development of CAC from that of sporadic CRC. This approach could potentially uncover novel signaling pathways and cellular roles in the inflammation-to-cancer transition.

## 5 Microbiota

**Figure 2:**
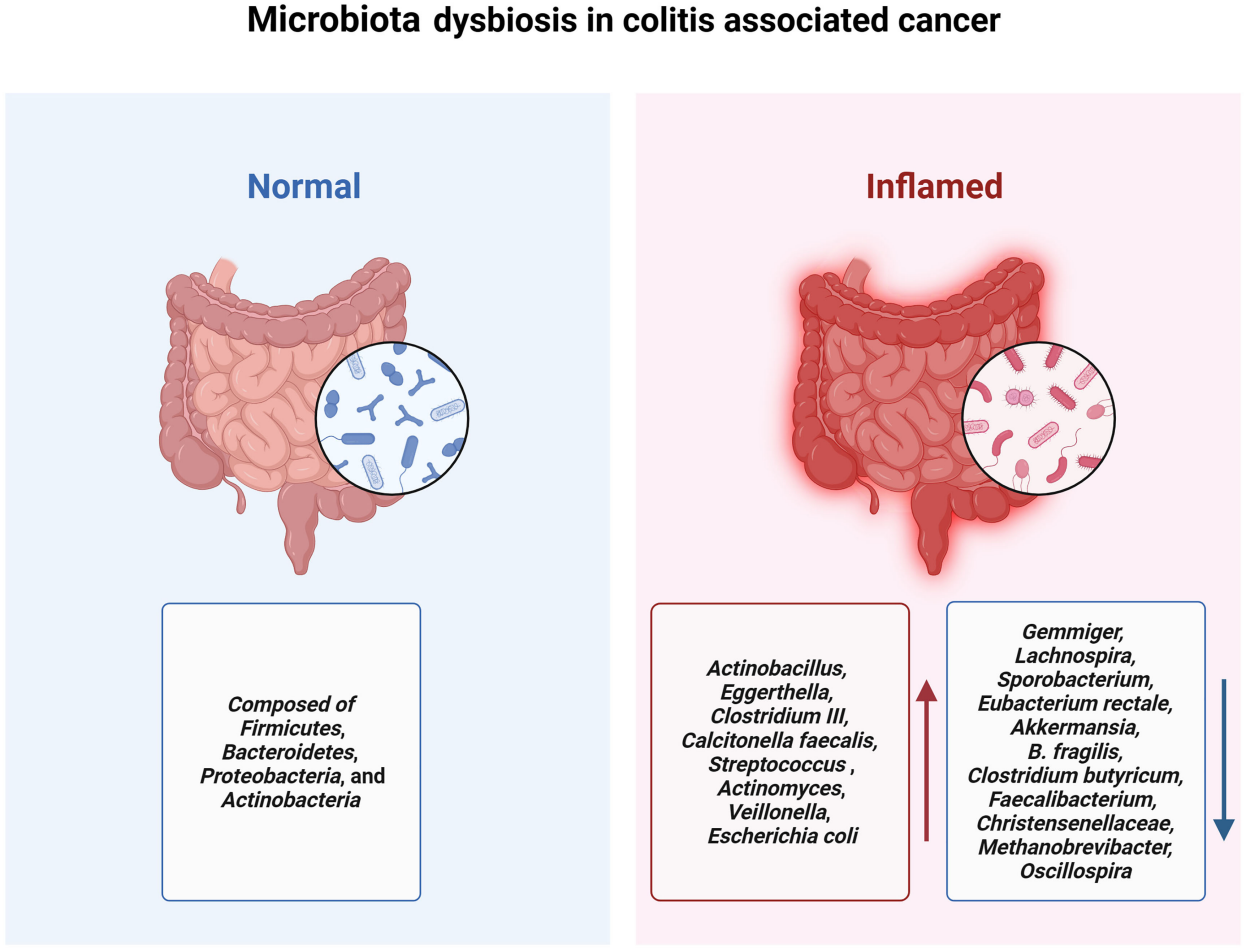
Patients with IBD experience a decrease in gut microbiota diversity accompanied by a reduction in probiotics and an increase in pathogenic bacteria, which contributes to the onset and progression of colitis-associated cancer.

The human and animal gut microbiome encompasses a diverse array of microorganisms, including bacteria, archaea, fungi, viruses, and multicellular parasites (115). The intestinal bacteria is predominantly composed of four bacterial phyla: *Firmicutes*, *Bacteroidetes*, *Proteobacteria*, and *Actinobacteria (* 116). The human gut microbiome is increasingly recognized for its role in IBD, It is primarily characterized by an increase in pathogenic bacteria coupled with a decrease in beneficial bacterial populations (**Figure 2**) (117, 118).

A study from Canada used 16S sequencing to compare the gut microbiota among individuals with CD (n=20), UC (n=19), and healthy controls (n=23). It was observed that microbial diversity in patients with IBD was significantly reduced compared to that in the healthy control group. And the microbial abundance of *Actinobacillus, Eggerthella, Clostridium III, Calcitonella faecalis, and Streptococcus* was significantly elevated, whereas the abundance of *Gemmiger, Lachnospira, and Sporobacterium* was significant decrease in all disease groups (119). A systematic review which included 48 studies in the analysis revealed elevated levels of *Actinomyces*, *Veillonella*, and *Escherichia coli* in patients and a diminished abundance of beneficial microbiota such as *Eubacterium rectale* and *Akkermansia (* 120).

Perturbations in the gut microbiota, along with concomitant immune dysregulation, play a central role in the pathogenesis of both IBD and CAC (121). In the initial stages of colitis-associated cancers, the gut microbiota employs lipopolysaccharide (LPS) to modulate monocyte-like macrophage (MLM) accumulation via a chemokine-dependent pathway. This process subsequently fosters a precancerous inflammatory environment by the pro- inflammatory immune cells activation that facilitates tumorigenesis (122). Notably, *Akkermansia* can alleviate colitis and curtail both IBD and CAC, potentially through the diminution of macrophages and CD8+ cytotoxic T lymphocyte (CTL) infiltration in the colon (123). A study recruited 144 age and gender-matched controls along with 41 patients with ulcerative colitis for gut microbiota testing from the Faroe Islands which have the highest incidence of IBD globally. In both groups, the *Akkermansia* genus was absent, shedding additional light on the potential susceptibility to inflammatory diseases in this high-risk population (124).

Short-chain fatty acids (SCFAs), especially butyrates, negatively regulate the inflammatory signaling pathway mediated by NLRP3 to inhibit the activation of macrophages and the secretion of pro-inflammatory mediators such as IL-18 and IL-1β, reducing intestinal inflammation levels and limiting CAC development. The abundance of beneficial bacteria, including *B. fragilis*, *Clostridium butyricum*, *Faecalibacterium*, *Christensenellaceae*, *Methanobrevibacter*, and *Oscillospira* which promotes the secretion of SCFAs significantly exhibited a significant decrease in the intestine of IBD patients during the CAC development process (125–130).

Considering these findings, the future development of therapies targeting opportunistic pathogenic or pro-inflammatory intestinal bacteria, or increasing the number of beneficial bacteria and metabolites like butyrates, represents a promising strategy for managing IBD. Such approaches could effectively alleviate colitis symptoms and prevent CAC in IBD patients.

## 6 Prevention of cancer associated with IBD

**Figure 3:**
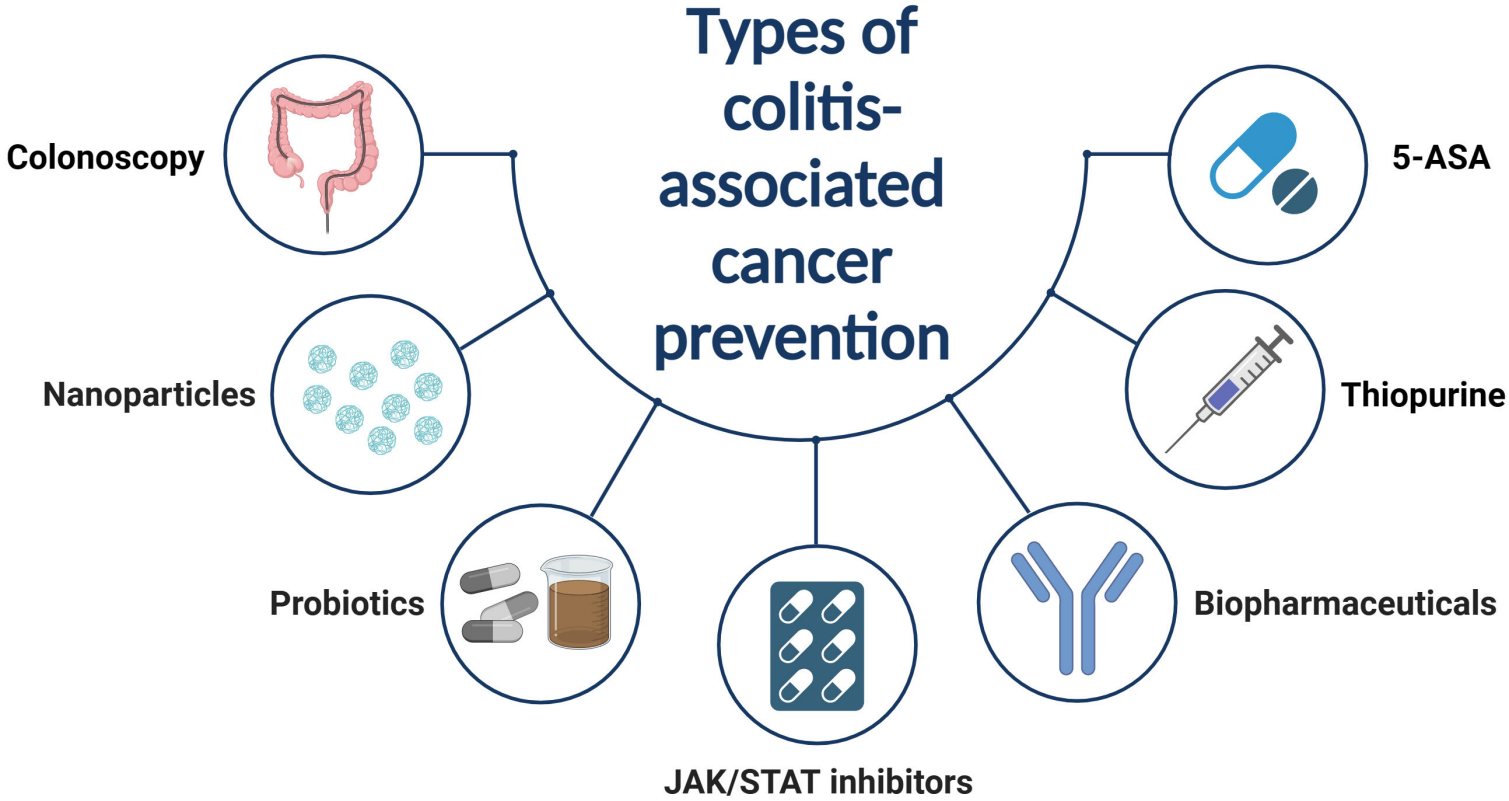
Traditional drugs for controlling IBD inflammation are believed to also prevent IBD from developing into CAC. However, the use of these drugs also carries the potential risk of causing other malignant tumors. Consequently, reasonable use of these drugs, combined with regular colonoscopy tumor screenings, is the most effective prevention measure.

In contrast to sporadic CRC, tumors associated with IBD typically evolve from inflammation to dysplasia, eventually leading to carcinoma. This progression is driven by continuous inflammatory stimuli (131). Consequently, the primary treatment strategies involve inflammation control and routine colonoscopic monitoring (132, 133). Current pharmacological interventions for IBD-associated cancer include traditional medications such as 5-aminosalicylic acid (5-ASA) and thiopurines, biopharmaceuticals, small molecule inhibitors, and some novel therapeutic approaches (**Figure 3**).

### 6.1 Conventional treatment

#### 6.1.1 5-ASA

5-ASA compounds, such as mesalazine, are frequently employed in managing inflammation in patients with IBD. Beyond its anti-inflammatory efficacy, 5-ASA has its potential in oncogenesis inhibition, particularly as it seems to decrease colorectal cancer incidence among long-term users (134). Microsatellite instability (MSI) is an early occurrence in the development of CAC and can be detected in chronically inflamed mucosa. Inhibiting MSI within the UC environment may aid in preventing CAC (135). The observed effect can be attributed to 5-ASA reducing IL-6-induced MSI, which contributes to its antitumor activity (136). This finding aligns with previous research on 5-ASA both *in vitro* and *in vivo (* 137, 138). A meta-analysis that compiled 9 studies showed a protective association in UC patients between the use of 5-aminosalicylic acid and CRC (OR=0.51) (139). Intrestingly, patients with UC experience more substantial benefits from 5-ASA compared to those with CD (140). 5-ASA does not provide a protective effect against small bowel adenocarcinoma in Crohn’s disease (141). 5-ASA may exhibit antitumor properties against colorectal tumors in IBD patients, but their protective efficacy against the progression to CRC in individuals with low-grade dysplasia appears to be constrained (57). Consistent with this study, another study indicates that the use of 5-ASA in IBD patients in the two years prior to a diagnosis of CAC does not have a preventive effect on CAC which suggests that the use of 5-ASA may not offer significant protection once dysplastic growth has already occurred in the later stages of the disease (142). In conclusion, current evidence suggests that the use of mesalazine as a chemopreventive treatment to reduce the risk of CAC in patients with UC is feasible and generally associated with relatively minor side effects. Furthermore, due to its mechanism of action, which involves inhibiting the synthesis of inflammatory mediators, reducing leukocyte activity, and enhancing the mucosal barrier, the effectiveness of this intervention appears to be enhanced when applied in the earlier stages of inflammation (143, 144).

#### 6.1.2 Thiopurines

Rapid induction and ensuring the maintenance of remission are fundamental to IBD treatment. In cases where remission is difficult to maintain with monotherapy of 5-ASA, it may be necessary to use immunomodulators (IMs) such as thiopurines for long-term remission (145). Moreover, thiopurines also have a chemopreventive effect on CAC. It works by inhibiting the activity of leukocytes (particularly T-cells) in the immune system, thereby slowing down the inflammatory response. Their primary mechanism of action involves inhibiting DNA synthesis and cell division, which effectively suppresses the proliferation and activity of immune cells (146). Local administration of thiopurine can alleviate colitis and enhance autophagy, reducing dysplasia and CAC induced by AOM/DSS in wild-type mice (147). And the present meta-analysis indicates that thiopurines have a chemopreventive effect on colorectal tumors in IBD patients, displaying a tendency towards diminishing the progression of these tumors (148). In patients with IBD, particularly those with an extended disease duration (>8 years), the use of thiopurine is correlated with a decreased risk of colorectal tumors, advanced neoplasia, and CRC (149). Medications such as IMs are pivotal for controlling inflammation in IBD, but they also potentially increase the risk of cancer (150). A study revealed a slightly increased risk of nonmelanoma skin cancers in IBD patients undergoing thiopurine treatment (151). In a long-term follow-up study of 19,486 IBD patients in France, those treated with thiopurines had a reduced risk (HR=0.28) of CRC in IBD patients compared to those who had never been treated with thiopurines. However, the risk of lymphoproliferative malignancies increased (HR=52.5) (152). In summary, thiopurines play a crucial role in the maintenance of remission and reduction of colorectal cancer risk in the treatment of IBD. However, they may also elevate the risk of certain specific cancer types, necessitating careful consideration and vigilant monitoring in clinical practice.

### 6.2 Biopharmaceuticals

The emergence of biologics, targeting mechanisms like leukocyte trafficking inhibition (anti-integrin antibodies) or inflammatory cytokine blockade (anti-tumor necrosis factor, anti-interleukin 12/23), has revolutionized our capacity to attain clinical remission and endoscopic healing, consequently reducing the onset of IBD-associated complications (153). A study utilizing a multi-center database in the United States (Explorys), encompassing 225,090 patients with Crohn’s Disease and 188,420 patients with Ulcerative Colitis, indicated that those treated with anti-TNF medications had a lower risk of developing CAC. The ORs for CD and UC were 0.69 and 0.78, respectively (154).

The activation of NF-κB pathway is crucial for the progression of CAC. The specific factors that directly induce NF-κB activation during the progression of CAC remain unclear. One potential mediator in the epithelial cells of patients with IBD is TNF-α, which is significantly elevated in the inflamed intestinal environment (155). And TNFR2 signaling in intestinal epithelial cells may directly contribute to the development of persistent CAC. This suggests that maintenance therapy with anti-TNF-α monoclonal antibodies can not only effectively control inflammation but also potentially halt the progression of CAC in long-term IBD (156). Under normal conditions, TNF-α plays a crucial role in immune system development and host defense against infectious agents. However, its role under pathological conditions is markedly different. In addition to causing dysregulation of the immune response, TNF-α can also contribute to inflammation or carcinogenesis, thus presenting a dual aspect to its function and its relation to NF-κB (157). The activation of the classical IKK-β/NF-κB pathway leads to increased transcription of genes including inflammatory mediators (COX-2, iNOS, TNF, and IL-6), proteases, and apoptosis inhibitors (BCL-XL, cIAPs, GADD45β, BFL1, and SOD2) (158–160). Studies have confirmed the involvement of these molecules in colitis-associated carcinogenesis. Infliximab’s mechanism in cancer prevention may be through the reduction of inflammatory mediators or the induction of apoptosis (161). Chronically elevated levels of TNF-α in tissues can also promote cancer growth, invasion, and metastasis (162).

In addition to cytokines, various growth and angiogenic factors, as well as matrix-degrading proteases like matrix metalloproteinases (MMP)-2, MMP-3, and MMP-9, play significant roles in tumorigenesis, invasion, and metastasis (163). Matrix metalloproteinases, which can be released by pro-inflammatory cytokines such as TNF-α and IL-1β, are crucial in tissue remodeling and destruction. Notably, MMP-9 is the most highly expressed protease in colonic inflammatory tissues (164). In experiments, involving mice treated with infliximab, there was a significant reduction in the expression and activity of MMP-9 and MMP-11, as well as β-catenin. This reduction led to decreased tumor occurrence in the AOM/DSS animal model (165).

Considering the therapeutic efficacy and drug resistance, a combination therapy approach is proposed. Combining Infliximab with immunosuppressants (such as thiopurine or methotrexate) can improve the pharmacokinetics of Infliximab. Although combination therapy represents a compromise treatment strategy, which can improve the pharmacokinetics, it still carries some risk of cancer development. A meta-analysis that included four observational studies, involving a total of 261,689 patients, showcased an increased risk of lymphoma in IBD patients administered with anti-TNFα agents, either as monotherapy or in conjunction with thiopurines (166). Additionally, the combination of Infliximab and azathioprine increases the risk of infections and malignant tumors. New biologics, such as Vedolizumab and Adalimumab, also elevate the risk of skin cancer when used together (167).

But some studies present alternative perspectives, several studies suggest that anti-TNFα therapy does not correlate with an increased malignancy risk in IBD patients. It is important to note that these studies do not provide information on malignancy risk beyond a treatment duration of one year, leaving potential long-term risks undetermined (168). For all cases of malignancies 'during treatment, it is essential to adopt a multidisciplinary approach involving gastroenterologists, dermatologists or oncology specialists, for direct and open communication about balancing IBD treatment with malignancy management (169).

### 6.3 Small molecule drugs

The JAK/STAT pathway has been demonstrated to be involved in the pathogenesis of IBD. Blocking this pathway can inhibit various pro-inflammatory cytokines, reducing intestinal inflammation. Tofacitinib, an orally administered small molecule drug, primarily inhibits JAK1 and JAK3. Clinical studies have confirmed that tofacitinib can induce and maintain remission in UC (170).

Studies have shown that polymorphonuclear cells (PMN)-derived reactive oxygen species (ROS) from oxidative bursts play a crucial role in inducing MSI in colorectal cells. Furthermore, PMN-derived cytokines, including IL-8, IL-6, and TNF-α, contribute to mucosal frameshift mutations (171). while JAK inhibitors can reduce mutations in intestinal epithelial cells by decreasing the release of cytokines from these cells (172). Therefore, both the removal of ROS and inhibition of cytokine signaling pathways by JAK inhibitors may prevent cancer progression in UC. PMNs not only produce ROS but also secrete a range of cytokines (e.g., IL-8, IL-6, and TNF-α), chemokines, and growth factors (173). These cytokines are elevated in both active UC and Crohn’s disease. In patients with CAC, IL-6 can alter DNA methylation through DNMT1-induced hypermethylation of the SOCS3 promoter, leading to subsequent STAT3 hyperactivation (174, 175). The use of JAK inhibitors may inhibit these mutations and thereby reduce the risk of CAC.

The JAK inhibitor tofacitinib eliminates Microsatellite instability (MSI) induced by IL-6 or neutrophils, potentially delaying or preventing the progression of cancer in cases of colitis (171). Tofacitinib, while capable of inhibiting the development of CAC, also presents certain risks. In a clinical study, 598 patients received tofacitinib induction therapy for 8 weeks, while 541 patients were administered a placebo.The results indicates tofacitinib increased the overall rate of infections, the incidence of herpes zoster, and the occurrence of non-melanoma skin cancer in IBD patients (176). In another meta-analysis that included 82 studies and 66,159 patients who were treated with JAK inhibitors, researchers found that the risk of herpes zoster infection increased in patients with immune-mediated diseases treated with JAK inhibitors. However, there was no increase in the risk of malignant tumors and other complications (177). There is a need for large-scale, long-term cohort studies to adequately assess the impact of these medications on the risk of CAC.

### 6.4 Probiotics

The role of probiotics in preventing the inflammation-to-cancer transition in IBD is a significant area of study. Probiotics have been shown to influence the growth of beneficial gut bacteria that can modulate immune responses against cancerous growth. Thus, the application of probiotics opens new possibilities for therapeutic strategies in cancer prevention (178).

As the most common probiotic, *Lactobacillus* plays a pivotal role in maintaining the ecological balance of the intestinal flora and exerts a beneficial anti-inflammatory effect in IBD and CAC. Acting symbiotically with the host, it helps maintain the immune microenvironment of the intestinal mucosa, limiting the over-activation of inflammatory signals and aiding patients in managing the inflammatory response in the gut. Anti-tumor effects were observed with *Lactobacillus bulgaricus*; its administration in an AOM/DSS-induced CAC mouse model suppressed mean tumor size and total tumor volume, significantly reducing pro-inflammatory cytokines, including IL-6, TNF-α, IL-1β, IL-17, and IL-23 (179). Similarly, a specific polysaccharide-peptidoglycan complex (PSPG) from *Lactobacillus casei Shirota* was shown to limit tumor growth by inhibiting the IL-6/STAT3 signaling pathway (180). The antiproliferative effect of *Lactobacillus helveticus NS8* on colon cells was more pronounced in the early stages of CAC, indicating its significant role in preventing tumorigenesis (181). Moreover, the fecal microbiota transplantation of *B. fragilis* has been proven effective in improving protection of intestinal epithelial damage caused by chronic inflammation and in preventing the development of colon tumors (125).

Due to the unique characteristics of different subspecies, the anti-inflammatory principles and functional components vary among them. Therefore, combining different strains of *Lactobacillus* to develop more effective probiotics, or using *Lactobacillus* in conjunction with other probiotics, could be a potent adjunctive therapeutic strategy for patients with IBD and related diseases. Recent research in this area has been extensive, yielding promising results. For instance, a probiotic combination of *Lactobacillus acidophilus*, *Bifidobacterium bifidum*, and *Lactobacillus rhamnosus* demonstrated potential chemopreventive effects by inhibiting tumor growth in AOM/DSS-induced CAC mice (182).

In addition to probiotics, the study of synbiotics, which combine probiotics and prebiotics or include the addition of vitamins and trace elements, has also garnered significant interest. Synbiotics enhance the physiological bacterial activity of probiotics and selectively and rapidly increase their population, making the function of probiotics more significant and long-lasting (183). A synbiotic comprising *Lactobacillus* 505 and *Cudrania tricuspidata* leaf extract has been reported to inhibit CAC development and reduce the incidence of colon tumors while significantly down-regulating pro-inflammatory cytokines, up-regulating tight junctions (TJs), and increasing pro-apoptotic factors such as p53, p21, and Bax in damaged colonic mucosa, thus demonstrating its therapeutic value in CAC (184).

Although probiotics has shown numerous anti-inflammatory effects in mice, its clinical efficacy is still limited by challenges such as low viability and bioavailability during gastrointestinal transit. Research on *Ligilactobacillus salivarius* has led to the development of a new probiotic encapsulation method using layer-by-layer (LbL) approach, significantly enhancing its potential to alleviate colitis (185). Advanced gas shear technology and ion diffusion have been used to prepare colon-targeted core-shell hydrogel microspheres, extending the local residence time of the drug and potentially enhancing the bioavailability of probiotics (186). Therefore, developing effective methods for probiotics packaging is equally important.

### 6.5 Other approachs

Beyond traditional medications, in recent years, exosomes derived from plants have been discovered to possess the capability to deliver drugs specifically to the intestinal tract, offering substantial potential in treating intestinal diseases. These exosomes boast numerous advantages, including excellent biocompatibility, non-toxicity, low immunogenicity, targeted delivery, an extended duration of drug action, high production capacity, and the ability to cross the blood-brain barrier (187). Studies indicate that for conditions like IBD and CAC, a variety of chemical and nucleic acid drugs can be efficiently transported to the site of intestinal inflammation via plant-sourced exosomes, helping to reduce inflammation or inhibit gene expression (188, 189). Such as nanoparticles derived from edible ginger, GDNPs 2, which reduce acute colitis, enhance intestinal repair, and prevent chronic colitis and CAC (190). In experiments, the nonsteroidal antiinflammatory drugs (NSAIDs) aspirin can also exert its protective effect against CAC through the immunomodulatory actions on macrophages and CD8+ T cells (191). Apart from drug-related treatments, regular and repeated colonoscopy with biopsies is considered the most effective method, with annual monitoring recommended for high-risk patients. Specifically, these high-risk individuals include those with extensive colitis with severe active inflammation, first-degree relatives diagnosed with CRC before the age of 50, those with concomitant Primary Sclerosing Cholangitis, and patients found with dysplasia within the past 5 years (192).

To summarize, optimized inflammation management, coupled with regular endoscopic monitoring and neoplastic screening are effective in preventing IBD-associated malignancies. But academic discussions still lack a definitive consensus regarding the potential cancer risks associated with the prolonged usage of these drugs. It is encouraging to note that, in addition to traditional therapies, there are now emerging novel drugs and treatment approaches that have shown promising efficacy in the prevention of CAC. And delineating and balancing their role in preventing IBD-associated malignancies is a critical matter that demands immediate attention. There is a urgent call for broader prospective and experimental research in this field.

## 7 Conclusion

Chronic inflammation resulting from IBD increases the annual incidence of various tumors in patients, encompassing both gastrointestinal and extragastrointestinal malignancies. The transformation from inflammation induced by IBD to carcinogenesis results from interplay among immune cells, gut microecology, and signaling pathways. Notably, various immune cells, exhibit dual roles during the various stages of inflammation and tumor development. In the initial inflammatory stage, these cells such as M1 macrophages, Th1 cells, and CD8 cells, exacerbate inflammation, which is counteracted by the anti-inflammatory actions of M2 macrophages,Th2 and Foxp3+ Treg cells. However, once the tumor microenvironment is established, these pro-inflammatory cells that initially promoted inflammation now act to suppress tumor cell viability by enhancing tumor immunity. Conversely, the anti-inflammatory cells then facilitate tumor immune evasion and progression. In addition, during the interactions among immune cells, changes in the gut microbiota of IBD patients, along with the activation of certain cancer signaling pathways in intestinal epithelial cells, also have been pivotal in the development of IBD-associated cancers. The protective effect of 5-ASA against CAC appears well-proved, though its early use during the initial stages of inflammation is crucial. The impact of other drugs, including IMs and anti-TNFα, as well as other novel drugs, needs better assessment of their impact on decreasing CAC risk and side-effects in long-term use through large prospective cohort studies. Furthermore, future research should focus on a deeper understanding of the key pathogenic pathways and molecular mechanisms of inflammatory-cancer transformation in IBD patients to facilitate the development of new treatment methods and targets.

## Author contributions

ML: Conceptualization, Funding acquisition, Supervision, Writing – review & editing. HZ: Investigation, Visualization, Writing – original draft. YLS: Writing – original draft. CL: Investigation, Writing – review & editing. CH: Funding acquisition, Supervision, Writing – review & editing. SW: Funding acquisition, Supervision, Writing – review & editing. QL: Supervision, Writing – review & editing. YS: Supervision, Writing – review & editing.
